# Comparative Insights on IL-5 Targeting with Mepolizumab and Benralizumab: Enhancing EGPA Treatment Strategies

**DOI:** 10.3390/biom15040544

**Published:** 2025-04-08

**Authors:** Mayu Shiomi, Ryu Watanabe, Ryuhei Ishihara, Sayaka Tanaka, Takashi Nakazawa, Motomu Hashimoto

**Affiliations:** 1 Department of Rheumatology, Osaka Saiseikai Nakatsu Hospital, Osaka 530-0012, Japan; 2 Department of Clinical Immunology, Osaka Metropolitan University Graduate School of Medicine, 1-4-3, Asahi-machi, Abeno-Ku, Osaka 545-8585, Japan; 3 Department of Pathology, Osaka Metropolitan University Graduate School of Medicine, Osaka 545-8585, Japan

**Keywords:** benralizumab, eosinophil, eosinophilic granulomatosis with polyangiitis, interleukin-5, mepolizumab

## Abstract

Eosinophilic granulomatosis with polyangiitis (EGPA) is a necrotizing vasculitis characterized by extravascular granulomas and eosinophilia in both blood and tissues. Eosinophils, which play a critical role in the pathophysiology of EGPA, require interleukin (IL)-5 for maturation in the bone marrow and migration to tissues. Glucocorticoids and immunosuppressants have been the cornerstone of treatment; however, their side effects have imposed a significant burden on many patients. Mepolizumab, an antibody that binds to and neutralizes IL-5, demonstrated efficacy in controlling disease activity in EGPA in the MIRRA trial conducted in 2017. In 2024, benralizumab, an IL-5 receptor alpha antagonist, was shown to be non-inferior to mepolizumab in efficacy against EGPA in the MANDARA trial. Both drugs were originally used for severe asthma and have benefited EGPA by reducing eosinophil counts. Due to differences in pharmacological structure and pharmacokinetics, the degree of eosinophil suppression varies between the two agents, and recent studies suggest that they may also affect inflammatory and homeostatic eosinophils differently. This review summarizes the latest insights into the pathophysiology of EGPA, highlights the similarities and differences between the two drugs, and discusses future treatment strategies for EGPA based on current clinical unmet needs, including drug selection.

## 1. Introduction

Eosinophilic granulomatosis with polyangiitis (EGPA), first reported in 1951 by Dr. Churg and Dr. Strauss, is one of the types of antineutrophil cytoplasmic antibody (ANCA)-associated vasculitis that affect small- to medium-sized vessels [1]. Traditional treatments for EGPA have primarily consisted of immunosuppressive therapies centered on glucocorticoids (GC) and cyclophosphamide. However, despite achieving disease activity control, relapse rates remain notably high following dose reduction or discontinuation of these medications. In cases with poor prognosis (five-factor score ≥ 1), the relapse rate was significantly higher in the GC monotherapy group compared to the group receiving combination therapy with cyclophosphamide [2]. Furthermore, severe adverse effects associated with long-term use of these treatments, such as infections and malignancies, cannot be overlooked [3,4]. These challenges underscore the urgent need to develop molecularly targeted therapies with fewer side effects, based on the underlying disease mechanisms. 

In 2017, the efficacy of mepolizumab, an interleukin-5 (IL-5) inhibitor, was demonstrated for EGPA in the MIRRA trial [5]. This drug not only controlled disease activity in EGPA but also enabled a reduction in GC use [5]. Subsequently, mepolizumab was approved for use in refractory and relapsing EGPA and has since become widely utilized. Furthermore, in 2024, benralizumab, an IL-5 receptor inhibitor, was shown to be non-inferior to mepolizumab in terms of efficacy for EGPA in the MANDARA trial, leading to its approval for use in this condition [6].

The introduction of IL-5-targeted therapies has significantly advanced the treatment strategy for EGPA. This review provides an overview of the role of eosinophils and IL-5 in the pathophysiology of EGPA, incorporating the latest research findings. Furthermore, it organizes the existing evidence on mepolizumab and benralizumab in asthma and EGPA, while summarizing the pharmacological characteristics, similarities, and differences between these two agents. The goal of this review is to offer insights into the optimal utilization of these therapies based on their distinct features.

## 2. Pathophysiological Differences in EGPA Based on ANCA Status: The Roles of Eosinophils and IL-5

**Figure 1:**
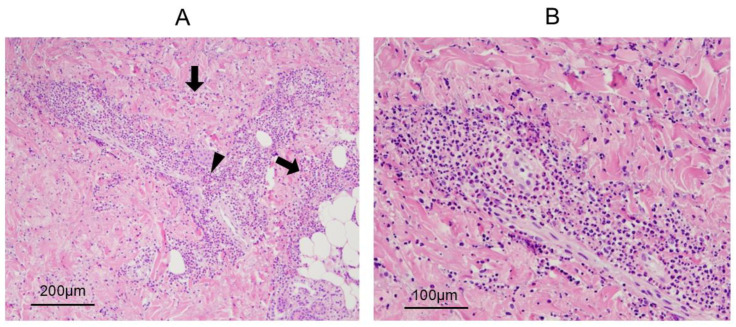
Skin biopsy findings with hematoxylin and eosin staining. (**A**) Eosinophilic infiltration is observed not only around the blood vessels (arrowhead) but also extensively within the collagen fibers and interstitial tissue (arrows). (**B**) Eosinophil-predominant inflammatory cell infiltration is evident within the vascular wall.

The typical progression of EGPA can be broadly categorized into three stages. It begins with a prodromal phase characterized by asthma and eosinophilic sinusitis, followed by a phase of peripheral blood and tissue eosinophilia. Ultimately, it progresses to the vasculitic phase, which is pathologically marked by extensive eosinophilic infiltration (Figure 1), leading to systemic organ damage including mononeuritis multiplex, purpura, and renal impairment [4,7].

The myeloperoxidase (MPO)-ANCA positivity rate in EGPA is approximately 30–40%, and differences in genetic background, pathophysiology, and organ involvement have been reported based on ANCA status (Table 1) [8,9,10,11]. In ANCA-positive cases, *HLA-DQ* has been identified as a risk allele, sharing a genetic background with MPO-positive ANCA-associated vasculitis. Conversely, ANCA-negative cases are characterized by mutations in *IRF1*/*IL5*, and *GPA33*, which are primarily associated with impaired airway mucosal barrier function. 

Reflecting these genetic backgrounds, ANCA-positive EGPA is predominantly characterized by neutrophil-driven vasculitis, with a higher likelihood of organ involvement such as glomerulonephritis and cutaneous purpura. On the other hand, in ANCA-negative EGPA, the vasculitic phase is thought to be primarily mediated by eosinophilic inflammation, leading to a greater propensity for eosinophilic myocarditis and eosinophilic gastroenteritis. The detailed mechanisms underlying these differences remain unclear; however, recent studies have increasingly elucidated the role of eosinophils in the pathophysiology of EGPA.

### 2.1. Pathophysiology of Eosinophilic Inflammation

The well-known roles of eosinophils include their involvement in allergic reactions and immune responses to parasitic infections. However, it has been revealed that eosinophils are present in tissues such as the skin, lungs, and gastrointestinal tract at levels approximately 100 times higher than in peripheral blood. Within these tissues, they contribute to maintaining homeostasis, including tissue repair and the lipid and glucose metabolism, and are even implicated in the suppression or promotion of malignant tumors [12,13,14,15].

In conditions such as eosinophilic gastrointestinal disorders, hypereosinophilic syndrome (HES), and EGPA, the hallmark of eosinophilic inflammation is degranulation, during which eosinophils release cytotoxic granules. Eosinophils store at least four types of granule proteins in their cytoplasm, each of which plays a role such as antimicrobial activity or the activation of Type 2 (T2) inflammation [16,17]. The mechanisms of eosinophil degranulation can be broadly categorized into two types: those involving cell lysis and those that do not [18]. In the organs of EGPA patients, a form of programmed eosinophil cell death known as eosinophil extracellular trap cell death (EETosis) has been observed. This process involves the release of eosinophilic granules, which are thought to contribute to the development of vasculitis [19].

### 2.2. The Multifaceted Role of IL-5 in Eosinophil Biology and EETosis

**Figure 2:**
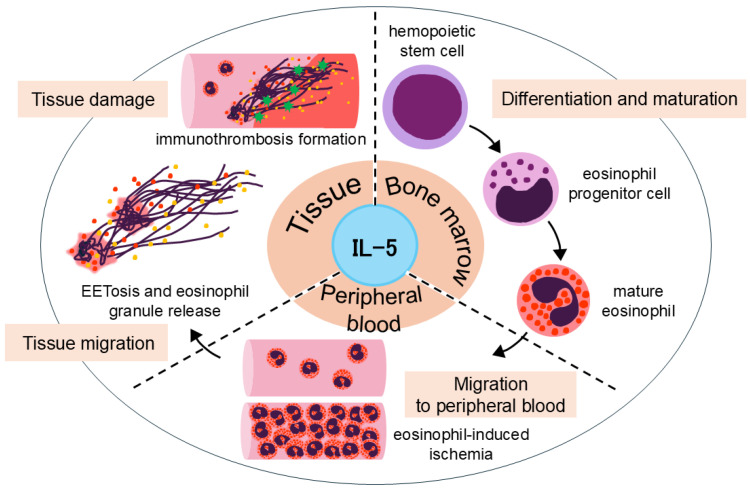
The role of IL-5 in the pathophysiology of EGPA. Interleukin-5 (IL-5) promotes eosinophil differentiation and maturation in the bone marrow and facilitates its migration into peripheral blood. In peripheral blood, excessive eosinophils can contribute to eosinophil-induced ischemia. Recent studies suggest that upon migrating into tissues, eosinophils become activated by IL-5 and other stimuli, leading to the induction of eosinophil extracellular trap cell death (EETosis). This process results in the release of galectin-10 and eosinophil granules, driving eosinophilic inflammation and tissue damage. Moreover, EETosis has been implicated in immunothrombosis formation, further contributing to EGPA pathogenesis.

IL-5 is a cytokine of critical importance that exerts multifaceted effects on the life cycle of eosinophils (Figure 2). IL-5 contributes to eosinophil differentiation and maturation in the bone marrow, migration to peripheral blood and tissues, and the prolongation of eosinophil survival [11,20,21,22].

Notably, IL-5 stimulation, along with other factors, plays a key role in inducing EETosis, potentially leading to the release of galectin-10 from the eosinophil cytoplasm. Galectin-10 levels have been shown to correlate with disease activity in EGPA and are positively associated with IL-5 [19]. Furthermore, the administration of mepolizumab in patients with severe asthma has been shown to reduce serum galectin-10 levels [23]. These findings suggest that IL-5-mediated eosinophil activation triggers a cascade involving EETosis and galectin-10 release. Moreover, the net-like structures formed during EETosis have been shown to promote platelet adhesion, implicating their role in immunothrombosis formation [24].

Based on the above molecular and biological findings, targeting IL-5 as a therapeutic strategy is highly rational from a pathophysiological perspective and is now regarded as an indispensable approach in the treatment of EGPA.

## 3. Mechanism of Action of Mepolizumab and Benralizumab

IL-5R is expressed not only on eosinophils but also on basophils, mast cells, and CD34+ progenitor cells [25,26,27]. The IL-5Rα subunit specifically binds to IL-5, while the βc subunit is involved in signal transduction [25,28]. There are three monoclonal antibodies targeting IL-5: mepolizumab, reslizumab, and benralizumab.

Mepolizumab, a monoclonal antibody against IL-5, inhibits IL-5 from binding to the IL-5 receptor α (IL-5Rα) on the eosinophil surface, thereby suppressing eosinophil proliferation. It has a high affinity for IL-5 and does not interfere with the biological activity of other cytokines [29].

Similarly, reslizumab, another IL-5 monoclonal antibody, shares a comparable mechanism of action with mepolizumab. It is approved for the treatment of severe eosinophilic asthma in the United States and Europe [29,30,31], but not in Japan. Since this review focuses on the comparison between mepolizumab and benralizumab, which is approved for EGPA, a detailed discussion of reslizumab falls beyond the scope of this article.

In contrast, benralizumab is a humanized monoclonal antibody targeting IL-5Rα. Unlike mepolizumab and reslizumab, which block IL-5 itself, benralizumab directly binds to IL-5Rα, preventing IL-5 from interacting with its receptor. Its most notable pharmacological feature lies in its glycosylation modification. Specifically, the Fc region of benralizumab, particularly the CH2 domain, has been engineered to lack fucose, enhancing its ability to bind strongly to eosinophils via FcγRIIIa receptors (also known as CD16) on natural killer (NK) cells. This modification amplifies antibody-dependent cellular cytotoxicity (ADCC) activity by 10–100 times [32,33,34].

**Figure 3:**
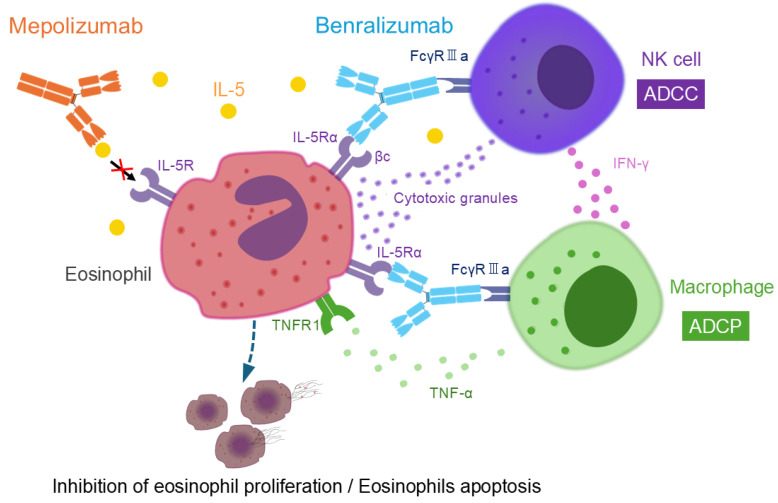
Differences in the mechanisms of action of mepolizumab and benralizumab on eosinophils. Mepolizumab is a humanized monoclonal antibody with high affinity and specificity for Interleukin-5 (IL-5). It inhibits the binding of IL-5 to its receptor on eosinophils, thereby suppressing eosinophil proliferation. Benralizumab, a humanized monoclonal antibody, binds specifically to IL-5 receptor α via its Fab fragment, blocking the interaction between IL-5 and its receptor. The Fc region of benralizumab interacts with FcγRIIIa receptors on natural killer (NK) cells, promoting antibody-dependent cellular cytotoxicity (ADCC) by facilitating the release of apoptosis-inducing proteins such as granzymes and perforin. Additionally, benralizumab enhances antibody-dependent cellular phagocytosis (ADCP) via FcγRIIIa on macrophages. Activated NK cells release interferon-γ, stimulating tumor necrosis factor (TNF)-α secretion from macrophages. TNF-α binds to TNF receptor 1 (TNFR1) expressed on eosinophils, inducing eosinophil apoptosis and consequently enhancing ADCP.

Furthermore, benralizumab promotes antibody-dependent cellular phagocytosis (ADCP) through FcγRIIIa on macrophages. NK cells activated by benralizumab release interferon (IFN)-γ, which stimulates tumor necrosis factor (TNF)-α secretion from macrophages. TNF-α binds to TNF receptor 1 expressed on eosinophils, inducing eosinophil apoptosis and consequently enhancing ADCP. Through these immune cell interactions involving NK cells, macrophages, and eosinophils, benralizumab effectively depletes eosinophils, leading to their near-complete depletion [35]. (Figure 3). In in vitro experiments, benralizumab strongly promoted ADCC and ADCP; however, complement-dependent cytotoxicity activity was not observed, presumably due to insufficient activation of the complement system [35]. Importantly, in vitro experiments have shown that eosinophil granules such as eosinophil cationic protein and eosinophil-derived neurotoxin are not released during benralizumab-induced eosinophil apoptosis, supporting the safety profile of benralizumab [36].

## 4. Comparison of the Characteristics of Mepolizumab and Benralizumab in Asthma

### 4.1. Asthma Exacerbations and Lung Function

The IL-5 pathway has been proven effective in suppressing asthma exacerbations in clinical trials. In the MENSA trial, mepolizumab reduced the annual exacerbation rate by 53% [37]. Similarly, benralizumab demonstrated a 45% and 35% reduction in exacerbations in the SIROCCO and CALIMA trials, respectively [38,39] and reduced the annual exacerbation rate by 74% in the MIRACLE trial conducted in Asia [40]. Furthermore, the XALOC-1 trial, a large-scale, multinational, retrospective observational study investigating benralizumab in adults with severe eosinophilic asthma, showed that benralizumab was associated with a reduction in exacerbation rates even in patients with a prior history of omalizumab or mepolizumab treatment [41]. A real-world comparative study of benralizumab and mepolizumab demonstrated significant reductions in the Asthma Control Questionnaire (ACQ) scores and asthma exacerbation rates with both therapies, indicating comparable efficacy [42].

Blood and sputum eosinophil counts have been shown to significantly correlate with worsening forced expiratory volume in 1 s (FEV1), a key marker of asthma control [43,44]. Both therapies demonstrated significant improvements in FEV1 after 12 months of treatment; the median FEV1 increased from 59% to 74% with mepolizumab and from 63% to 72% with benralizumab [45]. In real-world clinical practice, no clinically meaningful differences have been observed between mepolizumab and benralizumab in terms of their effects on lung function.

### 4.2. Glucocorticoid-Sparing Effects

In the SIRIUS trial, mepolizumab reduced GC doses by 50% while maintaining asthma control [46]. Similarly, benralizumab demonstrated numerically superior GC-sparing capabilities, achieving a 75% reduction in GC doses in the ZONDA trial [47]. Additionally, in the PONENTE trial, 81.9% of patients treated with benralizumab were able to reduce their prednisolone dose to ≤5 mg, and 62.9% achieved GC-free status [48]. The GC-sparing effects of benralizumab have also been supported by the large-scale XALOC-1 study, in which 47.4% of patients with severe eosinophilic asthma who received benralizumab were able to completely discontinue GC therapy after 48 weeks [41].

In real-world clinical practice, the proportion of patients with severe T2-high asthma who achieved remission—defined as the absence of exacerbations, no requirement for oral corticosteroids, an Asthma Control Test score of ≥20, and an ACQ-6 score of <1.5—was 29% with mepolizumab and 44% with benralizumab [49].

Both agents demonstrate GC-sparing effects in asthma management. However, as no large-scale trials have directly compared their efficacy, it remains premature to determine their relative superiority. Large-scale comparative studies are needed to further elucidate the clinical impact of their GC-sparing effects in asthma management.

### 4.3. Airway Remodeling Effect

Airway remodeling is a key feature of severe eosinophilic asthma, driving persistent airflow limitation and disease progression. IL-5R is expressed on bronchial fibroblasts, and, importantly, its expression is significantly elevated in fibroblasts from asthma patients compared to those from healthy controls [50]. Mepolizumab has demonstrated significant improvements in airway remodeling markers after 12 months, including reductions in sub-basement membrane thickness, airway smooth muscle (ASM) layer thickness, and epithelial damage, indicating structural restoration [51,52]. Benralizumab, which depletes eosinophils via ADCC and ADCP, reduces ASM mass by targeting TGF-β1-expressing eosinophils but has minimal impact on myofibroblast numbers, reflecting differences in remodeling effects [53]. These findings underscore the potential of IL-5-targeted therapies to address airway remodeling in severe eosinophilic asthma, while highlighting the need for further research on their differential impacts and long-term outcomes.

### 4.4. Safety Profiles

Headache (20%) and injection site reactions (8%) are the most commonly reported adverse reactions associated with mepolizumab treatment, based on clinical trial data. In contrast, headache (8%) and pharyngitis (5%) are more frequently observed with benralizumab [29]. Notably, long-term studies of benralizumab have identified no new adverse reactions attributable to eosinophil depletion, further supporting its safety profile [54,55]. Both therapies have demonstrated acceptable safety profiles in long-term use, making them suitable for the management of severe eosinophilic asthma. Continued monitoring in real-world clinical settings is warranted to confirm these findings and ensure the safe application of these therapies in broader patient populations.

## 5. Efficacy of Mepolizumab and Benralizumab in EGPA

### 5.1. Clinical Trial Evidence for Mepolizumab

The MIRRA trial, a pivotal international Phase 3 study, evaluated the efficacy and safety of mepolizumab in 136 patients with relapsing or refractory EGPA. In this study, remission was defined as a Birmingham Vasculitis Activity Score (BVAS) of 0 and a GC dose of ≤4 mg. The results demonstrated a significant difference in remission rates at weeks 36 and 48 between the mepolizumab group and the placebo group [5]. The trial also confirmed the drug’s effectiveness in reducing relapses and enabling GC tapering. Regarding safety, although injection site reactions and headaches were observed in approximately 10% of patients receiving mepolizumab, no serious adverse events were reported [5].

However, the MIRRA trial excluded patients with organ/life-threatening EGPA, and the proportion of ANCA-positive patients was relatively low, at 10%. The inclusion criteria resulted in a study population with limited external validity. Moreover, the majority of patients were already receiving GC therapy at baseline, and these factors may have influenced the outcomes. In a subsequent post hoc analysis of the MIRRA trial, remission was achieved and relapse was suppressed regardless of ANCA status or disease severity [56]. Another post hoc analysis demonstrated that remission was achieved and GC-sparing effects were observed regardless of the number of relapses at baseline, disease duration, or baseline GC dose [57]. These findings suggest that the body of evidence supporting the efficacy of mepolizumab continues to grow.

Currently, mepolizumab is recommended as a remission induction therapy in combination with GC for relapsing or refractory EGPA, in the absence of active organ/life-threatening disease. Evidence supporting the efficacy of mepolizumab in severe EGPA remains insufficient. As a remission-maintenance therapy, it is recommended regardless of the presence of poor prognostic factors and is considered a first-line option, particularly for maintaining remission in relapsing EGPA [3,4]. Although the current evidence indicates that mepolizumab is effective regardless of ANCA status [4,56], ANCA status may be a relevant factor in guiding treatment decisions in clinical practice, particularly as more real-world data become available.

### 5.2. Long-Term Efficacy of Mepolizumab in Real-World Clinical Practice

In the MIRRA trial’s Long-Term Access Programme (LAP), the retention rate of mepolizumab treatment was 71% at up to 89 months [58]. Similarly, in the Open-Label Extension (OLE) of the MIRRA trial, the retention rate for up to 7.6 years was 68% [59], indicating a high level of tolerability.

In the OLE, the median GC decreased from 10.0 mg/day at the OLE baseline to 5.0 mg/day at the end of the study [60]. The GC-sparing effects of mepolizumab was also demonstrated in a separate study using LAP data from the MIRRA trial, with an observation period of up to 7.4 years. Notably, patients who had already received mepolizumab during the MIRRA trial and had been on treatment for a longer duration were able to reduce their GC dose earlier [61].

Long-term outcomes in real-world clinical settings are gradually being elucidated. In a two-year multicenter observational study conducted in Europe, mepolizumab demonstrated an improvement in BVAS and a glucocorticoid-sparing effects [62]. We previously demonstrated in a multicenter cohort study that the five-year drug retention rate of mepolizumab in 60 patients with EGPA was 78.7%, indicating high efficacy and tolerability [63]. Additionally, we compared the clinical course between the mepolizumab-treated group and the non-treated group. Propensity score matching was used to adjust for baseline characteristics of the two groups. The results showed that the BVAS at the last observation was significantly lower, and the GC dose was significantly reduced in the mepolizumab group. Although there was no significant difference in the rate of achieving GC-free status, the proportion of patients achieving a GC dose of ≤4 mg was significantly higher in the mepolizumab group. Moreover, the use of mepolizumab was associated with achieving a GC dose of ≤4 mg, whereas poor asthma control was identified as a factor associated with failure to achieve this dose reduction. These findings suggest that mepolizumab contributes to reduce disease activity and GC tapering in EGPA in real-world clinical practice [64].

In another study of EGPA patients who had received mepolizumab treatment for more than 48 weeks, an additional 96 weeks of long-term treatment resulted in further reductions in GC dose. This study also suggested that suppressing EGPA relapses may help prevent the progression of neurological symptoms associated with peripheral neuropathy, potentially improving patient outcomes [65]. These findings support the long-term efficacy and safety of mepolizumab in the treatment of EGPA. 

### 5.3. Clinical Outcomes of Benralizumab

An international Phase 3 trial (MANDARA trial) was conducted in patients with relapsing or refractory EGPA. Remission was defined in the same manner as in the MIRRA trial: BVAS of 0 and GC dose of ≤4 mg. There was no significant difference in remission rates at weeks 36 and 48 between the benralizumab group and the mepolizumab group [6]; however, benralizumab demonstrated a more potent reduction in eosinophil counts than mepolizumab. The proportion of patients achieving GC-free status during the 52-week observation period was 41% in the benralizumab group and 27% in the mepolizumab group, suggesting that benralizumab may have superior GC-sparing effects. In this trial, the most frequently reported adverse events were COVID-19 infections (benralizumab/mepolizumab: 21%/27%), headaches (17%/16%), and arthralgia (17%/11%). The incidence of serious adverse events was 6% in the benralizumab group and 13% in the mepolizumab group [6]. During the open-label extension phase of the MANDARA trial, the safety profile of benralizumab was consistent with that observed in the double-blind phase [66].

Based on the positive results of the MANDARA trial, benralizumab has been approved as an additional therapeutic option for EGPA. Given the study design, benralizumab is expected to be used in a manner similar to mepolizumab for remission induction therapy. However, definitive evidence regarding its efficacy in remission maintenance therapy has not yet been established. 

It is important to note, though, that the MANDARA trial had several important limitations. As in the MIRRA trial, patients with organ/life-threatening EGPA were excluded, and the sample size was limited to 140, resulting in restricted follow-up data. Furthermore, secondary endpoints were not adjusted for multiplicity, and the discussion section did not adequately address the methodological limitations of the trial. 

In the subgroup analysis of the MANDARA trial, no significant association was observed between ANCA status and the proportion of patients achieving remission; however, given the aforementioned limitations, further case accumulation and subsequent analyses are warranted.

In real-world clinical practice, benralizumab has been increasingly reported to effectively suppress disease activity, as measured by BVAS, while enabling GC dose reduction in patients with EGPA [67,68,69,70,71,72,73]. The accumulation of long-term clinical data on benralizumab will be essential for a better understanding of its efficacy and safety profile, particularly in diverse patient populations and under real-world clinical conditions. 

## 6. Optimizing the Use of Mepolizumab and Benralizumab: Tailoring Treatment Strategies

### 6.1. Rapid and Comprehensive Eosinophil Suppression by Benralizumab

Subgroup analyses from the MANDARA trial confirmed that benralizumab rapidly and significantly reduced blood eosinophil counts as early as the first week of treatment. This effect may surpass that of mepolizumab [74]. Proteomic analysis using randomized trial samples further demonstrated that benralizumab, compared to mepolizumab, suppresses pathways related to eosinophil activation and migration at an earlier stage. This finding suggests the potential for rapid inflammation control in T2 inflammatory diseases involving T2 cytokines (e.g., IL-4, IL-5, IL-13) [75].

Benralizumab (30 mg subcutaneous injection every four weeks) has been demonstrated to significantly reduce eosinophils in peripheral blood, bone marrow, and gastrointestinal tissues in patients with HES involving organ symptoms [76]. Furthermore, it has been reported that benralizumab achieves a greater reduction in sputum eosinophil counts compared to mepolizumab [77]. In contrast, while mepolizumab reduces peripheral blood eosinophil counts, its efficacy in reducing tissue eosinophils appears to be limited. Furthermore, its ability to reduce eosinophil progenitor cells in the bone marrow is considered less pronounced [78,79]. In real-world clinical settings, further case accumulation is warranted to determine how this rapid eosinophil depletion translates into tangible benefits for patients.

### 6.2. Glucocorticoid-Sparing Effects

In the MANDARA trial, the GC-free achievement rate was 41% in the benralizumab group compared to 27% in the mepolizumab group, suggesting that benralizumab may have superior GC-sparing effects [6,80]. Furthermore, a subgroup analysis of the MANDARA trial demonstrated that among EGPA patients using medium- or high-dose inhaled corticosteroids (ICS), the proportion of patients achieving GC-free status was significantly higher in the benralizumab group compared to the mepolizumab group [81].

In real-world clinical practice, benralizumab also led to greater reductions in GC use compared to mepolizumab, with a higher proportion of patients achieving complete discontinuation [69]. The superior eosinophil-suppressing effect of benralizumab is hypothesized to contribute to its enhanced GC-sparing effects; however, additional evidence is needed to validate this hypothesis.

### 6.3. Eosinophil Depletion and Immune Homeostasis

It is important to emphasize that complete depletion of eosinophils is not always advantageous in treatment. Eosinophils have traditionally been understood as inflammatory subtypes (inflammatory eosinophils: iEos) involved in inflammatory diseases such as asthma. However, recent studies have identified a novel eosinophil subtype in the peripheral blood of asthma patients, known as homeostatic eosinophils (hEos), which contribute to the maintenance of immune homeostasis [14,82,83]. iEos and hEos exhibit distinct gene expression profiles, with iEos being highly IL-5 dependent, whereas hEos is relatively less dependent on IL-5. Since benralizumab exerts its effects through immunological mechanisms, it fully depletes both iEos and hEos, regardless of their IL-5 dependence. In contrast, mepolizumab effectively reduces IL-5-dependent iEos while partially preserving hEos. This approach facilitates the restoration of eosinophil balance to a state closer to that of healthy individuals. This unique property of mepolizumab enables symptom improvement in asthma and EGPA while maintaining a balance between inflammation suppression and immune homeostasis, underscoring its significant therapeutic advantage. 

With regard to the immunological changes induced by mepolizumab, a comprehensive analysis of bioactive mediators in the serum of patients with EGPA demonstrated that proteins involved in the Th17/innate lymphoid cells (ILC) 3 axis, angiogenesis, and adhesion molecule pathways were suppressed following mepolizumab treatment [84]. These findings suggest that mepolizumab may exert effects beyond the IL-5–eosinophil axis. However, the potential long-term effects of these changes remain unclear, highlighting the need for further fundamental research into the immunological effects of IL-5 biologics, including benralizumab.

### 6.4. Eosinophil Depletion and Its Potential Link to Infections

Eosinophils play a critical role in immune responses against bacteria, viruses, and fungi [12], and the potential link between eosinophil suppression and an increased risk of infection remains a highly important subject of discussion. In patients with GC-dependent severe asthma, most respiratory infections during benralizumab treatment were non-eosinophilic. Specifically, the increased incidence of infections did not correlate with peripheral blood or airway eosinophil counts but was significantly associated with elevated neutrophil counts in sputum [85,86]. This is a highly intriguing finding, especially when considered in the context of the reported dysfunction of NK cell activity under immunosuppressive conditions. 

In the context of viral infections, IL-5 transgenic mice exhibit eosinophilia accompanied by reduced Toll-like receptor (TLR)7 expression in the lungs. In a TLR7-deficient asthma model, decreased interferon production and an increased rhinovirus load were observed [87]. These findings suggest that IL-5 inhibition may positively influence the immune response against rhinovirus. Conversely, a recent study investigating the role of mast cells in viral infections demonstrated that IL-5-treated mast cells produced higher levels of interferons in response to viral challenge. Notably, the study also suggested that IL-5 may contribute to mast cell survival. These findings imply that anti-IL-5 therapy may potentially impair antiviral immune responses [88].

In a real-world study comparing the five-year long-term safety of mepolizumab and benralizumab in patients with severe eosinophilic asthma, no significant differences in safety profiles were observed between the two agents, and no increased risk of serious infections was reported [89]. Furthermore, a meta-analysis of randomized controlled trials evaluating infection-related adverse events suggested that IL-5 biologics may reduce the incidence of serious bacterial infections, pneumonia, and influenza [90]. Taken together, the current evidence remains insufficient to determine whether eosinophil suppression is ultimately beneficial or detrimental with respect to infection risk, underscoring the need for further research, including case accumulation and mechanistic studies. 

### 6.5. Eosinophil Suppression and Malignancy Risk

Eosinophils have been implicated in cancer, exerting not only direct antitumor effects through the release of cytotoxic granule proteins but also indirect antitumor effects through interactions with immune cells such as CD8⁺ T cells and macrophages. Conversely, eosinophils have also been suggested to contribute to the formation of an immunosuppressive tumor microenvironment, potentially promoting tumor progression [12,91]. These observations indicate that the role of eosinophils in cancer is heterogeneous and may depend on tumor type and microenvironmental context.

The impact of anti-IL-5 biologics on malignancy risk, as well as treatment strategies for patients with EGPA who develop malignancies, remains critical for clinical practice. However, clear evidence addressing these concerns is currently lacking.

At present, there is no conclusive evidence that treatment with mepolizumab or benralizumab increases the risk of malignancy [89,92,93]. Continued accumulation of case reports and long-term safety data is strongly warranted.

### 6.6. NK Cell Function and the Efficacy of Benralizumab: Implications for Anti–IL-5 Biologics and Immunosuppressant Use

Human NK cells are classified into two major subsets based on the relative expression of CD56 and CD16: CD56brightCD16− and CD56dimCD16+ [94,95]. CD56dimCD16+ NK cells differentiate from CD56brightCD16− NK cells and constitute 90% of NK cells in peripheral blood. This subset exhibits high cytotoxic activity, contains abundant perforin, granzyme, and cytotoxic granules, and has the ability to induce ADCC.

The therapeutic efficacy of benralizumab may depend on NK cell function. It has been suggested that patients with impaired NK cell function due to concomitant use of immunosuppressants (ISs) may experience limited treatment effects. In patients with severe asthma, responders to benralizumab demonstrated preserved ADCC function, whereas non-responders showed reduced IFN-γ secretion from NK cells and impaired ADCC function compared to responders [96]. Indeed, in vitro experiments showed that dexamethasone administration to NK cells resulted in reduced IFN-γ expression. This suggests a potential decrease in ADCC activity and eosinophil apoptosis mediated by macrophages [96]. Furthermore, in patients with GC-dependent severe asthma who exhibited an insufficient response to benralizumab, a reduction in NK cell counts and a decreased proportion of CD16-positive NK cells, which are responsible for inducing ADCC activity, have been observed [85].

Recent studies on severe eosinophilic asthma have revealed an imbalance between mature CD16-positive NK cells and immature CD16-negative NK cells in asthma patients compared to healthy controls. After 24 months of benralizumab treatment, an increase in mature NK cells, specifically the CD56dimCD16bright subset, was observed, which correlated with changes in FEV1 and GC use [97]. The mechanisms by which benralizumab alters NK cell subsets remain unclear, and further research is warranted.

Regarding the combination of anti–IL-5 biologics and ISs, a post hoc analysis of the MIRRA trial demonstrated that mepolizumab was effective regardless of baseline IS use, including agents such as azathioprine and mycophenolate mofetil [57]. In contrast, a subgroup analysis of the MANDARA trial suggested that the remission rate with benralizumab appeared to be higher in patients without baseline IS use than in those receiving ISs at baseline [6]. However, given the small sample size and the major limitations previously noted in the MANDARA trial, this finding should be interpreted with caution. Currently, there is no established evidence regarding the concomitant use of these two IL-5–targeted agents with ISs. Nevertheless, as such combinations may have clinical implications for patient outcomes in real-world practice, the generation of further evidence is critically needed.

### 6.7. Effects of Benralizumab on Basophils

IL-5R is expressed on basophils and mast cells, suggesting that benralizumab can also exert effects on these immune cells, a characteristic not observed with mepolizumab [25,26,27]. In real-world clinical practice, benralizumab has been shown to reduce not only eosinophils but also basophils and mast cells [69,98,99]. Notably, studies evaluating basophils in severe eosinophilic asthma have demonstrated that benralizumab reduces basophil counts without inducing degranulation, highlighting its potential therapeutic utility [100]. 

Human basophils express the thymic stromal lymphopoietin receptor (TSLPR), which contributes to the pathogenesis of T2 inflammation [101,102,103]. Basophils can also be induced by IL-3, and TSLP-induced basophils exhibit distinct gene expression profiles compared to IL-3-induced basophils, suggesting heterogeneity [103,104]. Specifically, TSLP-induced basophils predominantly express genes related to fatty acid metabolism and cell adhesion molecules, whereas IL-3-induced basophils express genes associated with TNF-α signaling and the maturation of monocytes and dendritic cells [103].

A study comparing the characteristics of basophils before and after six months of benralizumab treatment in patients with severe eosinophilic asthma found that the baseline basophil count in asthma patients was significantly higher than that in healthy subjects. Interestingly, after benralizumab administration, the number of basophils expressing IL-3R and IL-5R significantly decreased, whereas there was no significant effect on basophils expressing TSLPR [105]. These findings suggest the presence of basophil subsets that respond differently to benralizumab; however, the clinical significance of this differential response remains unclear. Moreover, the roles of basophils and mast cells in the pathogenesis of EGPA remain largely unclear, emphasizing the need for further investigations to elucidate their contributions.

### 6.8. Cardiac and Thrombotic Implications of IL-5–Targeted Therapy in EGPA

Cardiac involvement is relatively common in EGPA and has been associated with a poor prognosis [4]. The role of eosinophils and IL-5 in cardiac pathophysiology remains inconclusive and debated. A study in the general population demonstrated that lower peripheral blood eosinophil counts were associated with an elevated short-term risk of mortality due to heart failure and coronary artery disease [106]. Furthermore, experimental studies using mouse models have suggested that IL-5 may promote tissue repair and improve cardiac function following myocardial infarction by inducing M2-type macrophages through eosinophil activation [107]. In contrast, clinical case reports have indicated that mepolizumab contributed to improved cardiac function and enabled GC tapering in patients with eosinophilic myocarditis associated with HES or EGPA [108].

In addition, EGPA has also been reported to be associated with both arterial and venous thrombosis. Interestingly, this thrombotic risk does not appear to be related to traditional cardiovascular risk factors such as hypertension, hyperlipidemia, or smoking, but rather to increased disease activity [109]. In real-world clinical practice, a low incidence of thrombotic events has been observed in patients with EGPA treated with mepolizumab. Patients who developed thrombosis had higher BVAS at the onset and were more likely to exhibit cardiac involvement compared to those without thrombosis [110]. Furthermore, a real-world study comparing the cardiovascular adverse effects of anti-IL-5/IL-5Rα antibody therapies with those of the anti-immunoglobulin E (IgE) antibody omalizumab found that cardiovascular events were reported less frequently with anti-IL-5/IL-5Rα therapies [111]. These findings suggest that thrombotic events in EGPA may be associated with underlying vasculitic activity, and that treatment with IL-5-targeted agents may help mitigate this risk. However, the current evidence is limited, and further accumulation of clinical data is warranted.

### 6.9. Paradoxical Arthritis Under Anti–IL-5 Biologics

Arthralgia has been reported as a paradoxical reaction associated with IL-5 antibody therapies [112,113]. Recent studies have identified a subset of eosinophils known as regulatory eosinophils, which play a role in suppressing inflammation in arthritis [114]. Interestingly, in this study, rheumatoid arthritis patients with comorbid asthma experienced a recurrence of arthritis following mepolizumab treatment. In the MANDARA trial, joint pain was observed in 17% of patients treated with benralizumab and 11% of those treated with mepolizumab [6]. However, progression to rheumatoid arthritis or inflammatory arthritis as a side effect of IL-5 antibody therapies is very rare [115]. The long-term adverse effects of these therapies remain largely unknown, underscoring the need for further case accumulation and studies.

### 6.10. Immunogenicity and Clinical Impact of Anti-Drug Antibodies 

A systematic review and meta-analysis investigating the incidence of anti-drug antibodies (ADA) in biologics approved for asthma treatment reported an ADA incidence of 8.35% for benralizumab and 3.63% for mepolizumab [116]. In the MANDARA trial, ADAs and neutralizing antibodies were observed in 9% and 1.5% of patients in the benralizumab group, respectively. However, these antibodies did not have a significant impact on achieving remission [6]. Similarly, a study evaluating the immunogenicity of mepolizumab in 915 patients across five major clinical trials (DREAM, MENSA, SIRIUS, COSMOS, and COLUMBA) detected ADAs in less than 1–9% of patients, with half of these cases being transient. Furthermore, neutralizing antibodies were identified in only a small number of patients, and no effect on treatment efficacy or adverse events was observed. No differences in adverse events or therapeutic outcomes were noted between ADA-positive and ADA-negative patients [117].

These findings suggest that there is no evidence that ADAs or neutralizing antibodies associated with either benralizumab or mepolizumab affect their efficacy and safety. Nonetheless, further data collection is necessary to comprehensively evaluate their clinical significance.

The unique characteristics of mepolizumab and benralizumab described above are summarized in Table 2.

## 7. Proposals for Drug Selection and Decision-Making in Clinical Practice

Thus far, we have outlined and summarized the respective characteristics and distinctions between mepolizumab and benralizumab. In this section, we explore their practical application and differential use in clinical settings.

In eosinophil-associated disorders, the number of affected organs has been reported to increase with rising eosinophil counts [118]. In real-world practice, 54.1% of EGPA cases have been diagnosed during the eosinophilic phase, and 30.7% during the vasculitic phase [119]. Early initiation of anti-IL-5 biologics during the prodromal or eosinophilic phase may be considered to prevent progression to the vasculitic phase.

The therapeutic efficacy of IL-5 biologics may vary among individuals. Regarding the relationship between blood eosinophil counts and treatment response, it has been reported that EGPA patients with higher baseline eosinophil levels are more likely to achieve remission following treatment with mepolizumab or benralizumab [69,70]. In a transcriptomic analysis of mepolizumab responsiveness in patients with EGPA, those who exhibited a favorable clinical response demonstrated an upregulated expression of genes associated with AP-1 transcription factors and IL-1β signaling pathways [120]. This finding suggests that interindividual differences in gene expression profiles may underlie the heterogeneous responses to IL-5–targeted therapies. Continued advances in research will be essential for the implementation of precision medicine in EGPA.

With regard to the switching of biologic agents, several case reports have described patients with severe eosinophilic asthma who achieved improved disease control after transitioning from mepolizumab to benralizumab due to an inadequate response [121]. Furthermore, in cases where EGPA developed during treatment with anti–T2 inflammatory monoclonal antibodies, the majority of patients were reinitiated on anti-T2 agents following the induction of remission. It has been reported that switching from benralizumab to mepolizumab, or vice versa, was associated with sustained remission without relapse in EGPA [122]. However, these observations are based on a limited number of cases, and high-quality evidence regarding the switching of anti–IL-5 biologics in EGPA remains insufficient. Further accumulation of clinical data and the establishment of robust evidence are warranted.

**Figure 4:**
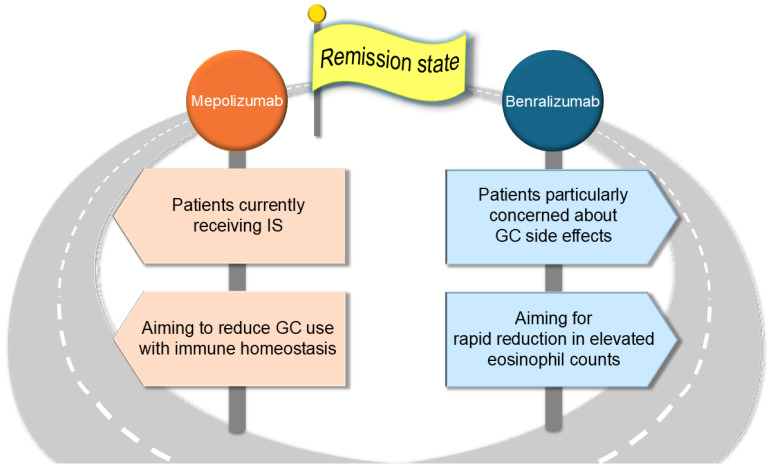
Hypothesis for the preferable selection of mepolizumab and benralizumab in EGPA treatment. Mepolizumab, unlike benralizumab, might be more effective in patients currently receiving ISs, where NK cell dysfunction is anticipated. Furthermore, mepolizumab may reduce inflammatory eosinophils while preserving homeostatic eosinophils, potentially maintaining immune homeostasis and promoting GC reduction. Conversely, benralizumab might rapidly reduce eosinophil counts in cases with extremely high levels. It may also reduce GC dependency in patients who are particularly concerned about GC-related side effects.

Based on the points discussed in this review, key considerations for the clinical use and differentiation of the two agents in real-world practice are summarized below. Mepolizumab might be effective even in patients currently receiving ISs, as its efficacy is less likely to be influenced by NK cell dysfunction. Furthermore, by selectively reducing inflammatory eosinophils while preserving eosinophil homeostasis, mepolizumab may contribute to long-term immune stability and facilitate GC tapering. In contrast, benralizumab may induce a rapid reduction in eosinophil counts, particularly in cases with markedly elevated eosinophil levels. Additionally, for patients with substantial concerns regarding GC-related adverse effects, benralizumab may help mitigate GC dependency (Figure 4).

Moving forward, it will be essential to accumulate evidence on how the differences between these two agents translate into clinical benefits for patients in real-world settings, and to develop a decision-making algorithm to guide their optimal use.

## 8. Exploring Alternative Therapeutic Targets Beyond IL-5 in EGPA

This review provides a comparative analysis of the pharmacological properties and clinical utility of mepolizumab and benralizumab. By systematically evaluating the latest scientific evidence, it provides practical insights to guide the optimal selection of IL-5 antibody therapies in real-world clinical settings. Furthermore, this review examines novel therapeutic target molecules identified through recent research beyond the IL-5 signaling pathway and discusses their therapeutic significance and potential for clinical application.

### 8.1. Proinflammatory Role of Charcot–Leyden Crystals

Charcot–Leyden crystals (CLCs) have long been recognized as a characteristic histopathological finding in eosinophilic inflammation. Recent studies have revealed that CLCs are produced during the process of EETosis [19]. The protein constituting CLCs, galectin-10, does not exhibit biological activity in its non-crystalline state. However, upon crystallization to form CLCs, it induces the production of inflammatory cytokines and amplifies T2 inflammatory responses [123]. Antibody therapies targeting and dissolving CLCs have been shown to suppress airway inflammation and IgE production [124].

Research on the regulatory mechanisms of EETosis has also progressed. An essential molecular mechanism in the EETosis is the function of peptidylarginine deiminase 4 (PAD4), which catalyzes histone citrullination. Experimental studies have demonstrated that inhibiting PAD4 suppresses EETosis [125]. Furthermore, in chronic eosinophilic rhinosinusitis, extracellular traps released during EETosis have been shown to degrade through the combined use of heparin and DNA-degrading enzymes [126].

The development of novel therapies targeting EETosis, a critical process in the pathogenesis of vasculitis in EGPA, is being actively pursued. The clinical application of innovative treatment strategies based on these fundamental research findings is highly anticipated.

### 8.2. IL-33: A Key Alarmin of ILC2 Activation

Group 2 ILC2 are rapidly activated by epithelial-derived alarmins such as IL-33 and TSLP, primarily releasing IL-5 and IL-13. IL-5 produced by ILC2 is essential for the recruitment of eosinophils to tissues and subsequent tissue damage [127,128,129,130]. A Phase 2 trial evaluated the efficacy of itepekimab, an IL-33-targeting therapy, in asthma [131]. Itepekimab improved asthma control, enhanced lung function, and demonstrated a favorable safety profile. In mouse studies, IL-33 was shown to induce and exacerbate EGPA in normal mice, independently of systemic eosinophilia [132]. IL-33 has gathered attention as an alarmin that plays a crucial role in the pathogenesis of EGPA, and its clinical application as a novel therapeutic target is highly anticipated.

### 8.3. NMU-NMUR1 Pathway

Neuromedin U (NMU) is a neuropeptide that acts on ILC2 to promote T2 inflammation. Its receptor, neuromedin U receptor 1 (NMUR1), is specifically expressed on ILC2 [125]. In patients with asthma, NMU activates ILC2 more rapidly than IL-33, highlighting the importance of the NMU–NMUR1 pathway in the early activation of ILC2 [133]. However, it has been shown that NMUR1 expression is suppressed under high-dose ICS treatment, suggesting that the NMU–NMUR1 pathway may be insufficiently functional in severe asthma or GC-resistant conditions. Future studies are required to comprehensively evaluate the role of NMUR1 and explore its potential as a novel therapeutic target.

### 8.4. HIF-1α/Glycolysis Axis in ILC2 Metabolism

Hypoxia-inducible factor-1α (HIF-1α) plays a central role in the metabolic regulation of ILC2s and is a critical factor in the pathogenesis of allergic airway inflammation [134]. In ILC2s stimulated by IL-33, upregulation of HIF-1α, a key regulator of glycolysis, was observed [135]. The administration of glycolysis inhibitors significantly suppressed ILC2 proliferation and the production of IL-5 and IL-13. Furthermore, in patients with asthma, decreased activity of the HIF-1α/glycolysis signaling pathway was positively correlated with the suppression of ILC2 function. These findings suggest that the HIF-1α/glycolysis signaling pathway plays a pivotal role in regulating ILC2 responses in allergic inflammation and may represent a novel therapeutic target for EGPA.

## 9. Conclusions

This review highlights the molecular roles of eosinophils and IL-5 in EGPA pathophysiology and summarizes the mechanisms and clinical benefits of the anti-IL-5 therapies mepolizumab and benralizumab. The approval of benralizumab has expanded the treatment options for EGPA, underscoring the need for personalized therapy based on disease activity, immunological phenotype, and comorbidities. Future research should focus on diverse patient populations and the optimization of treatment strategies.

## Figures and Tables

**Table 1:** Comparison of clinical and pathophysiological features between MPO-ANCA positive and negative EGPA.

	MPO-ANCA Positive EGPA	MPO-ANCA Negative EGPA
Genetic background	*HLA-DQ*, *HLA-DR*	*IRF1*/*IL5*, *GPA33*
Pathophysiology	Neutrophil-mediated vasculitis	Eosinophilic inflammation
	- ANCA binding to primed neutrophils induces ROS production, NET formation, and endothelial injury, leading to vasculitis.	- IL-5 promotes eosinophil proliferation, activation, and degranulation, resulting in cytotoxicity and tissue inflammation.
Organ involvement	Peripheral neuropathy glomerulonephritis cutaneous purpura	Eosinophilic myocarditis eosinophilic pneumonia eosinophilic gastroenteritis

**Table 2:** Summary table: distinguishing features of mepolizumab and benralizumab in the management of EGPA.

	Mepolizumab	Benralizumab
Mechanism of action	- Humanized anti-IL-5 monoclonal antibody - Blocks IL-5 signaling, inhibiting eosinophil proliferation	- Humanized monoclonal antibody targeting IL-5 receptor α - Depletes eosinophils via ADCC and ADCP
Effects on eosinophils and other immune cells	- Reduces eosinophils in peripheral blood, possibly in tissues and bone marrow - Selectively suppresses iEos - May help maintain immune homeostasis	- Almost completely depletes eosinophils in blood, bone marrow, and tissues - Reduces basophils without degranulation - Efficacy may depend on CD56dimCD16bright NK cells
Clinical trials	- MIRRA Trial (2017) - 300 mg subcutaneous Q4W	- MANDARA Trial (2024) - 30 mg subcutaneous Q4W
Efficacy in RCTs	- Superior to placebo in achieving remission and relapse control - Reduced glucocorticoid requirements	- Non-inferior to mepolizumab - Higher potential for glucocorticoid-free achievement (41% vs. 27%)
Safety profiles	- Common: injection site reactions, headache (~10%) - No severe adverse effects reported	- Common: COVID-19-related events - Slightly lower severe adverse events than mepolizumab (6% vs. 13%)
ADA incidence	~3.6%	~9%
Preferred patient profile	- Patients currently receiving IS	- Patients particularly concerned about GC-related side effects
Primary treatment objective	- GC tapering while preserving immune balance	- Rapid eosinophil reduction and minimizing GC toxicity

## Data Availability

Not applicable.
